# Forest Fire Detection Algorithm Based on Improved YOLOv11n

**DOI:** 10.3390/s25102989

**Published:** 2025-05-09

**Authors:** Kangqian Zhou, Shuihai Jiang

**Affiliations:** 1 School of Mechanical and Electronic Engineering, Nanjing Forestry University, Nanjing 210037, China; zhoukangqian@njfu.edu.cn; 2 Institute of Intelligent Control and Robotics Technology, Nanjing Forestry University, Nanjing 210037, China

**Keywords:** YOLOv11n, forest fire, lightweight, attention mechanism, dynamic upsampling operator

## Abstract

To address issues in traditional forest fire detection models, such as large parameter sizes, slow detection speed, and unsuitability for resource-constrained devices, this paper proposes a forest fire detection method, FEDS-YOLOv11n, based on an improved YOLOv11n model. First, the C3k2 module was redesigned using the FasterBlock module, replacing C3k2 with C3k2-Faster in both the Backbone network and Neck section to achieve a lightweight model design. Second, an EMA attention mechanism was introduced into the C3k2-Faster module in the Backbone, replacing C3k2-Faster with C3k2-Faster-EMA to compensate for the accuracy loss in small-object detection caused by the lightweight design. Third, the original upsampling module in the Neck was replaced with the lightweight dynamic upsampling operator DySample. Finally, the detection head was improved using the SEAM attention module, replacing the original Detect head with SEAMHead, which enables better handling of occluded objects. The experimental results show that compared to YOLOv11n, the proposed FEDS-YOLOv11n achieves improvements of 0.9% in precision (P), 1.9% in recall (R), 2.1% in mean precision at IoU 0.5 (mAP@0.5), and 2.3% in mean precision at IoU 0.5–0.95 (mAP@0.5–0.95). Additionally, the number of parameters is reduced by 21.32%, GFLOPs are reduced by 26.98%, and FPS increases from 48.2 to 71.8. The FEDS-YOLOv11n model ensures high accuracy while maintaining lower computational complexity and faster inference speed, making it suitable for real-time forest fire detection applications.

## 1. Introduction

As a crucial component of the Earth’s ecosystem, forests protect biodiversity resources and serve as natural species and gene repositories [1]. Forests are closely related to human life, contributing in various ways—not only improving living environments but also providing significant economic value [2]. However, forest fires cause massive damage to the natural environment [3] and pose severe threats to human society. As a sudden and highly destructive natural disaster, forest fires are more challenging to extinguish and for rescue compared to ordinary fires [4]. Many major forest fire incidents result from a failure to suppress fires in their early stages, making the timely detection of forest fires crucial.

Before the widespread adoption of deep learning, forest fire monitoring primarily relied on recognizing fire targets in images based on flame color characteristics, shape features, motion properties, or texture information. In recent years, the continuous advancement of deep learning technology has led to revolutionary improvements in image recognition accuracy. Unlike traditional methods that rely on manually designed features, deep learning approaches automatically learn and extract features from large datasets through neural networks, reducing manual intervention and enabling more accurate and efficient object detection. Consequently, many researchers worldwide have proposed various deep learning-based forest fire detection methods. Two-stage object detection algorithms include R-CNN, Mask R-CNN [5], and Faster R-CNN [6]. These methods offer high detection accuracy for forest fires. However, they also suffer from high computational costs and slow inference speeds. These limitations make them unsuitable for real-time fire monitoring and difficult to deploy on resource-constrained devices. One-stage object detection algorithms, such as YOLO (You Only Look Once) [7] and SSD (Single Shot MultiBox Detector) [8], directly perform object classification and regression without requiring an additional region proposal step. As a result, they offer faster computation and inference speeds, making them suitable for real-time fire monitoring and deployment on resource-limited devices. Although YOLO-based methods generally have slightly lower detection accuracy than two-stage methods, their performance can be improved through model enhancements such as attention mechanisms, ensuring effective fire detection. Wu et al. [9] employed SSD for fire detection and proposed a new structure, tiny-yolo-vocl, which improved fire detection accuracy. Zhao et al. [10] proposed an improved deep learning algorithm, Fire-YOLO, based on YOLOv3. This model utilized EfficientNet for feature extraction from input images, enhancing feature learning. By expanding the feature extraction network across depth, width, and resolution dimensions, Fire-YOLO improved small-object recognition while reducing model parameters. However, it still faced issues such as low detection accuracy and difficulty in detecting partially occluded objects. Li et al. [11] introduced MobileNetv3’s bottleneck structure into YOLOv5 to make the model more lightweight, incorporating depthwise separable convolutions. They also employed a multi-scale feature fusion strategy and integrated coordinate attention and the SPPF (Spatial Pyramid Pooling-Fast) module to enhance feature extraction and improve detection accuracy for extreme wildfires. Wang et al. [12] proposed a lightweight detector, Light-YOLOv4, for real-time flame and smoke detection. This model adopted MobileNetv3 as the Backbone network, utilized bidirectional cross-scale connections, and incorporated split convolutions to process channel and spatial regions separately. These modifications significantly reduced model parameters, striking a balance between detection performance and efficiency. Bahhar C et al. [13] combined a dual-weight YOLO architecture with a voting-integrated CNN system. If the CNN detected anomalies in an image, YOLO further localized smoke or flames, achieving promising detection results. Yin et al. [14] proposed a smoke detector based on Transformer and YOLOv5, which enhances global feature representation by integrating an improved pooling structure. The model improves weak smoke detection by incorporating additional detection heads and feature fusion mechanisms. To mitigate the sensitivity of IoU to small object localization, the NWDLoss function is introduced. Furthermore, a multi-receptive field module is employed to strengthen multi-scale feature extraction, making the model well-suited for complex smoke detection tasks. Building upon this foundation, several studies have further expanded the application scenarios of wildfire detection methods and introduced multimodal data to enhance detection capabilities. Seydi et al. [15] proposed a deep learning framework named Fire-Net, which integrates optical and thermal modalities from Landsat-8 imagery to detect active wildfires and biomass burning. By employing residual connections and depthwise separable convolutions, the model is capable of extracting more abstract and deeper features. Almeida et al. [16] introduced a novel lightweight convolutional neural network model capable of processing RGB images captured by UAVs or surveillance systems in real time on edge devices, enabling timely wildfire detection and early warning. This approach avoids cloud-based data transmission, making it suitable for edge computing environments, and demonstrates superior performance compared to existing methods. Furthermore, Almeida et al. [17] enhanced the original EdgeFireSmoke algorithm and proposed EdgeFireSmoke++, which integrates artificial neural networks with deep learning techniques to efficiently monitor forest fires through real-time video streams. The algorithm supports processing at 33 frames per second, making it well-suited for early warning applications in small-scale forest reserves and effectively compensating for the limitations of traditional monitoring approaches.

YOLOv11 [18], released by Ultralytics in September 2024, is a real-time object detection model that incorporates multiple improvements over previous YOLO versions, significantly enhancing overall performance. In forest fire detection, YOLOv11n demonstrates notable advantages such as high speed, precision, and strong small-object detection capabilities, making it well-suited for real-time monitoring and multi-scenario adaptability. To further improve the detection speed and accuracy of YOLOv11n, this paper proposes an enhanced forest fire detection algorithm: FEDS-YOLOv11n. The model improves detection speed through a lightweight network and enhancement modules. At the same time, it strengthens the ability to detect small objects while maintaining detection accuracy. First, the FasterBlock module is used to redesign the C3k2 module, replacing C3k2 with C3k2-Faster in both the Backbone and Neck sections for a more lightweight architecture. Next, an attention mechanism, EMA, is introduced into the C3k2-Faster module in the Backbone, replacing it with C3k2-Faster-EMA. Additionally, the original upsampling module in the Neck is replaced with a lightweight dynamic upsampling operator, DySample. Finally, the original Detect head is replaced with an improved detection head, SEAMHead, which incorporates the Separated and Enhancement Attention Module (SEAM) to enhance the model’s ability to handle occluded objects. To reduce the number of parameters and computational complexity, the model has been optimized with the goal of future deployment on embedded edge devices. Specifically, we plan to deploy the FEDS-YOLOv11n model on the NVIDIA Jetson Nano platform to further validate its real-time detection capability in resource-constrained environments.

## 2. YOLOv11 Algorithm

**Figure 1:**
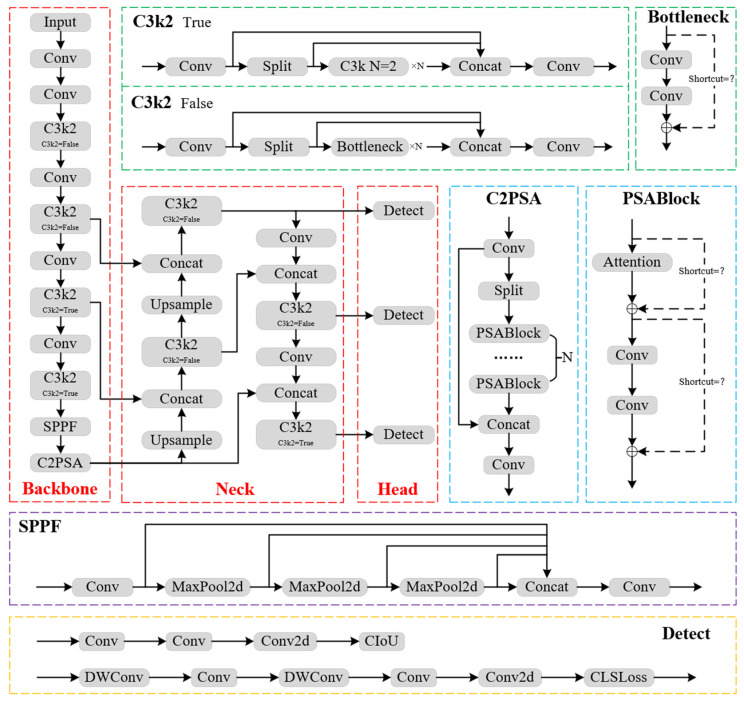
Network structure of YOLOv11.

Compared to YOLOv8, released by Ultralytics in January 2023, YOLOv11 introduces two novel feature extraction modules: C3k2 and C2PSA. The C2f modules in the Backbone and Neck sections of YOLOv8 are replaced with C3k2 modules in YOLOv11, and the C2PSA module is added after the SPPF module to further enhance the model’s ability to detect small objects. To improve small-object detection, YOLOv11 optimizes the feature fusion approach, enhancing multi-scale detection capability. The classification head incorporates two depthwise separable convolutions (DWConv), significantly reducing the number of parameters and computational complexity, making the detection head more lightweight. Through improved feature extraction modules and new training strategies, YOLOv11 achieves higher average precision than YOLOv8 while significantly reducing the model size, balancing detection accuracy and real-time performance. The network structure of YOLOv11 is illustrated in Figure 1.

YOLOv11 consists of three main components: Backbone, Neck, and Head. The Backbone extracts features through a series of convolutional layers and C3k2 modules, further enhancing feature representation with the SPPF and C2PSA modules. The Neck utilizes upsampling and cross-layer concatenation (Concat) to combine features at different scales. Finally, the Detect module in the Head performs object detection. The entire model design emphasizes multi-scale feature fusion to improve detection performance for objects of various sizes [19].

## 3. Materials and Methods

**Figure 2:**
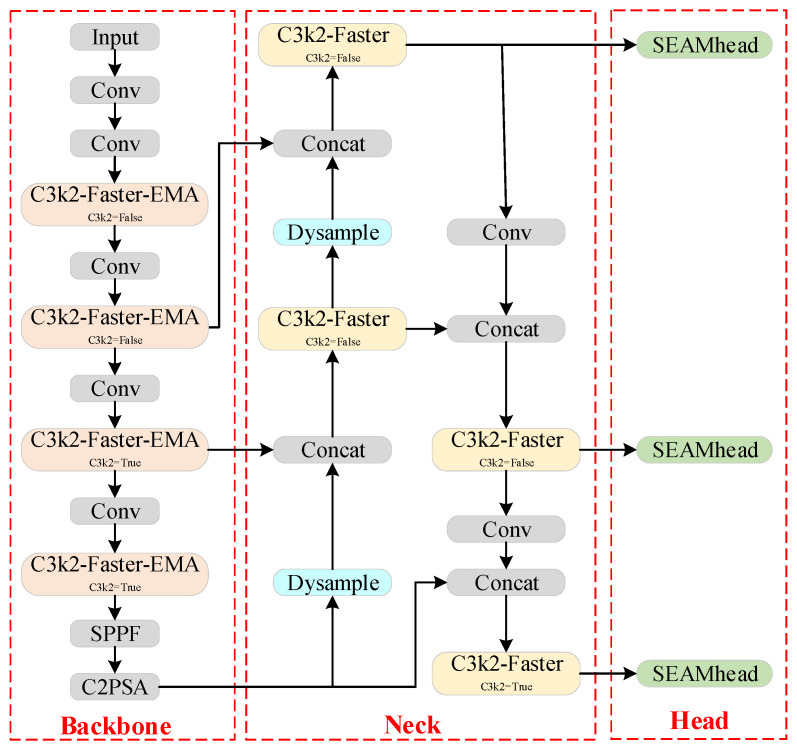
Structure of FEDS-YOLOv11n forest fire detection model.

Although YOLOv11n provides a lightweight version, processing high-resolution input images still requires significant computational resources. Additionally, the complex and dynamic nature of forest environments can impact the detection capability of YOLOv11n, reducing its accuracy. To ensure both detection precision and speed, this paper proposes the FEDS-YOLOv11n forest fire detection model based on YOLOv11n, as illustrated in Figure 2.

### 3.1. FasterBlock

**Figure 3:**
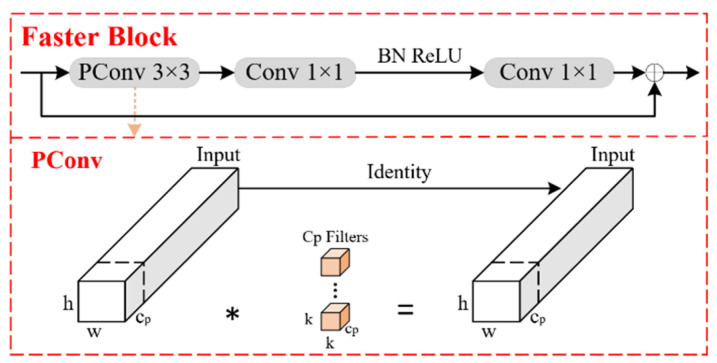
FasterBlock network structure.

Forest fire inspection robots operate under resource constraints, and due to the rapid spread of wildfires, they must detect fires in time to prevent further escalation. In the improved YOLOv11n model, the Bottleneck module in C3k2 is replaced with the FasterBlock module from FasterNet. This modification allows the model to achieve faster inference speed and lower computational complexity. It also enhances real-time detection capabilities while maintaining or even improving detection accuracy. The introduction of the FasterBlock module makes the model more suitable for deployment on resource-limited edge devices. FasterNet [20] is a lightweight and efficient network architecture designed primarily to reduce computational complexity and enhance inference speed while maintaining high accuracy, making it well-suited for real-time tasks. The core component of FasterNet is the FasterBlock module, an efficient feature extraction unit designed to reduce redundant computations and minimize channel overhead while preserving effective information representation. The network structure of FasterBlock is shown in Figure 3.

FasterBlock utilizes depthwise separable convolutions, significantly lowering computational complexity and reducing the number of parameters while still capturing rich spatial and channel information. Inside FasterBlock, a residual structure is implemented to ensure stable gradient propagation, addressing the vanishing gradient problem in deep networks. By incorporating a compression-expansion mechanism, FasterBlock first compresses and then expands the number of channels, reducing computation while retaining critical information. Additionally, FasterBlock employs the ReLU activation function and batch normalization, enabling the network to learn more complex feature representations efficiently. FasterBlock is efficient, lightweight, and capable of strong feature representation. It reduces the YOLOv11n model’s parameter count and computational complexity, while still preserving crucial information. This ensures training stability, enhances deep network performance, and improves the model’s ability to comprehend complex visual tasks.

### 3.2. Attention Mechanism: EMA

**Figure 4:**
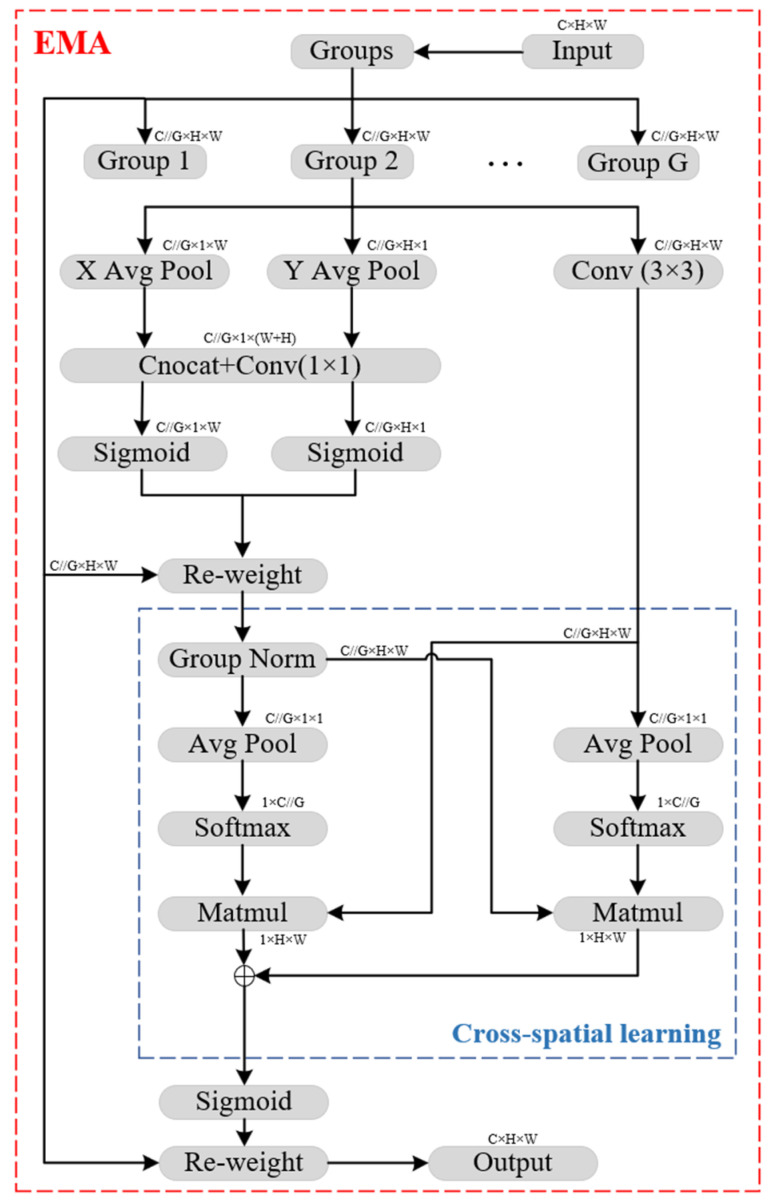
Network architecture of the EMA.

The complexity of forest environments and the challenges associated with small object detection pose significant difficulties for the YOLOv11n detection model. To enhance the model’s ability to detect small objects, an attention mechanism is introduced. Replacing the C3k2 module with C3k2-Faster reduces the model’s computational load and parameter count. This improves detection speed but may negatively affect detection accuracy. To address this issue, this paper integrates the Efficient Multi-scale Attention (EMA) mechanism [21] and replaces the C3k2 module in the Backbone with C3k2-Faster-EMA. Various attention mechanisms exist, among which EMA is a highly efficient multi-scale attention mechanism. EMA operates as a parallel attention mechanism, enhancing feature extraction robustness and efficiency while reducing computational complexity. This allows for improved model performance and processing speed. The structural model of EMA is shown in Figure 4.

In Figure 4, “g” represents the divided groups, “X Avg Pool” denotes one-dimensional horizontal global pooling, and “Y Avg Pool” represents one-dimensional vertical global pooling. The EMA attention mechanism first partitions the input feature map $X\epsilon R^{C\times H\times W}$ into G groups along the channel dimension, generating multiple sub-features to facilitate the capture of diverse semantic information. Each group can be represented as: $X=\left[X_{1},X_{2},\ldots ,X_{G-1}\right],\;{\;X}_{i}\epsilon R^{C//G\times H\times W}$. EMA then employs parallel subnetworks with large receptive fields to extract multi-scale spatial information. To compute attention weight descriptors, EMA combines three parallel branches: two 1 × 1 branches and one 3 × 3 branch. The 1 × 1 branches involve two 1D global average pooling operations, encoding information from two spatial directions. These two encoded feature representations are then concatenated along the vertical direction of the image and processed through a shared 1 × 1 convolution layer. The output of the 1 × 1 convolution is split into two vectors, each passed through a nonlinear Sigmoid function to establish a binomial distribution model. A simple multiplicative aggregation is applied to the two parallel 1 × 1 branches. The 3 × 3 branch utilizes a single 3 × 3 convolution kernel to capture multi-scale feature representations.

As a result, EMA encodes information across channels to adjust their relative importance while preserving precise spatial structural details. Additionally, EMA achieves a cross-spatial information aggregation strategy for feature interaction. Specifically, 2D global average pooling is applied to the 1 × 1 branch output, while the 3 × 3 branch output is directly adjusted to align with the corresponding dimensional structures before channel feature activation. To ensure computational efficiency, the Softmax function is applied to the output of the 2D global average pooling. By performing matrix dot product operations on the results from the previous parallel processing stages, an initial spatial attention map is generated. This approach efficiently integrates spatial information at different scales in the same processing stage. Similarly, global spatial information is embedded into the 3 × 3 branch using 2D global average pooling. Before the joint activation mechanism involving channel features, the output of the 1 × 1 branch is adjusted to match the corresponding dimensional configurations, yielding a second spatial attention map that retains precise spatial position information. Finally, the Sigmoid function is applied to the output feature maps in each group for further processing. The EMA attention mechanism leverages an exponential moving average (EMA) strategy, striking a balance between local feature aggregation and global information expansion. This makes it highly suitable for efficiently modeling complex contextual information in input data, particularly in scenarios requiring reduced computational complexity.

The EMA module combines channel and spatial information, preserving information along the channel dimension while reducing computational burden. This integration helps capture cross-channel relationships in feature representation while avoiding the loss of critical information due to channel dimension reduction, thus improving model performance. The EMA module adopts a multi-scale parallel sub-network architecture, consisting of one sub-network handling 1 × 1 convolutions and another handling 3 × 3 convolutions. This architecture effectively captures cross-dimensional interactions and establishes dependencies between different dimensions, enhancing feature representation capabilities. The parallel sub-network design in the EMA module facilitates feature aggregation and interaction, thereby improving the model’s ability to capture long-range dependencies. This design also avoids excessive sequential processing and deep network architectures, making the model both more efficient and effective.

### 3.3. DySample

**Figure 5:**
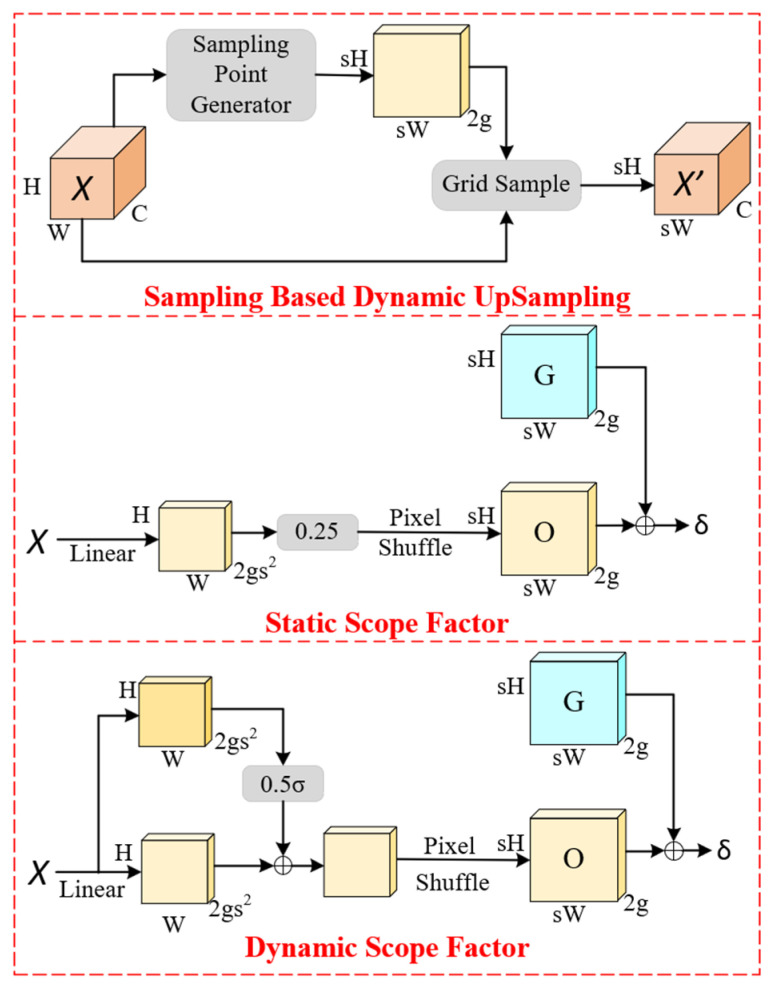
Dysample network structure.

In the original YOLOv11n model, the upsampling layers employ the UpSample function, which uses interpolation methods to upsample feature maps to higher resolutions. This approach increases the model’s computational load and parameter count, making it difficult to maintain a lightweight structure for forest fire detection. To address data imbalance issues in forest fire detection, enhance the model’s ability to detect small objects, and improve overall performance, this paper introduces the DySample upsampling module to replace the original upsampling mechanism. DySample is a recently proposed sampling optimization method in deep learning, particularly designed for semantic segmentation tasks [22]. It aims to overcome the limitations of conventional sampling strategies when handling class imbalance. The core idea of DySample is to dynamically adjust sampling weights for different pixels, allowing the model to focus more on challenging categories or regions during training. The DySample network architecture is illustrated in Figure 5.

Given a feature map X of size $C\times H_{1}\times W_{1}$ and a sampling set S of size $2\times H_{2}\times W_{2}$, where the first two dimensions represent the x and y coordinates, the grid_sample function uses the locations in S to resample X via bilinear interpolation, producing an output feature map X’ of size $C\times H_{2}\times W_{2}$. This process is defined as follows:

(1) $$X^{’}={grid}_{sample}(X,S)$$

Given an upsampling scale factor s and a feature map X of size C×H×W, a linear layer is used to generate an offset matrix O of size $2S^{2}\times H\times W$, where the input and output channel sizes are C and $2S^{2}$, respectively. Pixel Shuffling [23] is then applied to reshape O into size 2×sH×sW. The sampling set S is obtained by adding the offset matrix O to the original sampling grid G, omitting the reshaping operation. Finally, based on Equation (2), the sampling set is used to generate the upsampled feature map X’ of size C×sH×sW.

(2) O=linear(X)

(3) S=G+O

The Neck of the YOLOv11n network contains two UpSample layers, which are responsible for upsampling small feature maps from deeper layers of the Backbone to match the feature map sizes between deeper and shallower layers. In this study, we propose replacing the UpSample layers with DySample, a dynamic point-based upsampling method, to enhance the quality of upsampled features. This improves feature fusion in the Neck, while reducing the computational load and parameter count of the model.

### 3.4. SEAMhead

**Figure 6:**
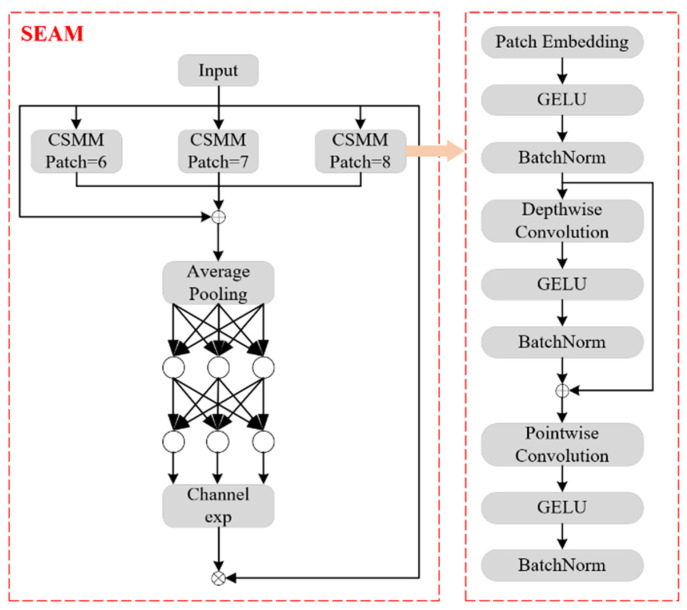
SEAM attention mechanism network structure.

To address feature misalignment, local aliasing, and key feature loss caused by occlusion, Yu et al. [24] proposed the Separated and Enhancement Attention Module (SEAM), a multi-head attention network designed to enhance multi-scale detection by emphasizing critical image regions and reducing background interference. The SEAM attention module is illustrated in Figure 6, where the left side presents the overall SEAM architecture, while the right side shows the Channel and Spatial Mixing Module (CSMM) structure. The CSMM extracts multi-scale features from different image regions and employs depthwise separable convolutions to learn correlations between the spatial and channel dimensions.

SEAM first applies depthwise separable convolutions with residual connections, performing convolution separately for each channel. This method reduces the number of parameters while learning the importance of different channels. However, it ignores inter-channel relationships. To compensate for this limitation, pointwise (1 × 1) convolutions are used to combine the outputs of different depth convolutions. Furthermore, a two-layer fully connected network fuses information across channels, strengthening inter-channel interactions. SEAM captures long-range dependencies in both spatial and channel dimensions. This enables it to learn the relationship between occluded and non-occluded objects, reducing the negative impact of occlusions.

The output logits of the fully connected layer undergo an exponential function transformation, mapping values from [0,1] to [1,e]. This exponential normalization provides a monotonic mapping, improving the model’s tolerance to positional errors. Finally, the SEAM output serves as attention weights, which are multiplied with the original feature maps to enhance the detection capability of the detection head and improve the model’s robustness to occlusions [25].

By integrating the SEAM module into the detection head of YOLOv11n, the model strengthens the association between occluded and non-occluded regions, thereby reducing the impact of occlusion on detection accuracy. Moreover, without significantly increasing computational cost, SEAM enhances inference speed and improves model robustness in complex environments.

## 4. Results

### 4.1. Dataset

**Figure 7:**
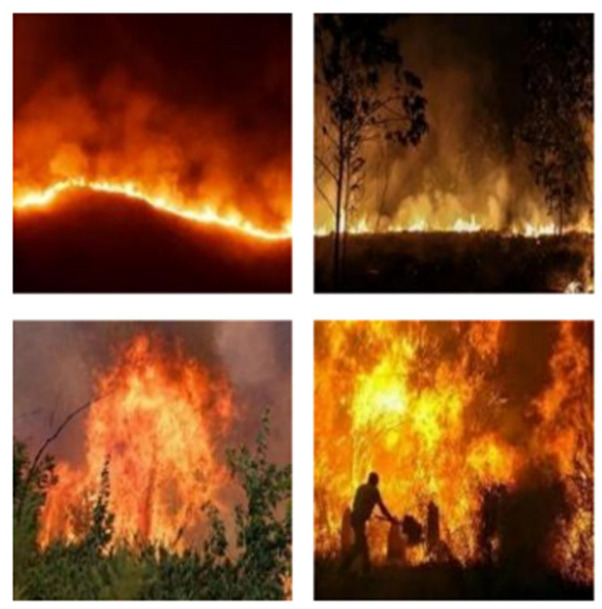
Example of a forest fire dataset.

In this study, the forest-fire-detection-qkpap_dataset from the Roboflow platform was selected. The images are in YOLOv11 format, and the annotations and configuration are provided in YOLOv11’s TXT and YAML formats. The dataset contains a total of 7514 images of forest fires, with 5262 images in the training set, 1502 images in the validation set, and 750 images in the test set. The ratio of the training, validation, and test sets is 7:2:1. An example from the forest fire dataset is shown in Figure 7.

### 4.2. Model Operating Environment and Parameter Settings

The training platform used for this experiment is an independent workstation running Windows 10 64-bit, with an Intel(R) Xeon(R) CPU E5-2695 v3 @ 2.30 GHz, NVIDIA GeForce RTX 3090 Ti GPU, and 24.0 GB of RAM. The Anaconda 3 virtual environment was configured for deep learning, with Python 3.10 as the programming language. The deep learning framework used is PyTorch 2.1.2, with CUDA 11.8. The input image resolution is 640 × 640 pixels. The Stochastic Gradient Descent (SGD) optimizer was used to update the model weights, with an initial learning rate of 0.01, a batch size of 64, and 300 iterations.

### 4.3. Evaluation Metrics

To evaluate the effectiveness of the model in object detection, the following metrics were chosen: precision (P), recall (R), mean average precision (mAP), mAP50, mAP50-95, parameters (Params), and GFLOPs (gigaflops). The calculation formulas are as follows:

(4) $$P=\frac{TP}{TP+FP}$$

(5) $$R=\frac{TP}{TP+FN}$$

(6) $$mAP=\frac{\sum AP}{N(class)}$$

where TP is the number of correctly detected targets, FP is the number of false positives, FN is the number of missed targets, P is precision, R is recall, AP is the area under the precision-recall curve, N(class) is the total number of classes, mAP50 is the mean AP when the Intersection over Union (IoU) is set to 0.50, mAP50-95 is the mean AP calculated at IoU values from 0.50 to 0.95 with an interval of 0.05. P and R are calculated when the IoU threshold is 0.50. GFLOPs measures the model or algorithm’s complexity. Params represents the model’s size. In general, smaller Params and GFLOPs indicate that the model requires less computational power and has lower hardware performance demands. Frames per second (FPS) is a critical metric for evaluating the performance of object detection models, as it reflects inference speed. In real-time forest fire monitoring scenarios, the standard for real-time performance is typically defined as processing at least 20 frames per second (FPS ≥ 20).

### 4.4. Experimental Results and Analysis

#### 4.4.1. Ablation Experiment

This study first used the FasterBlock module to lightweight the C3k2 module. Then, the EMA attention mechanism was added to the C3k2-Faster model to improve small object detection. The Backbone and Neck parts of the YOLOv11 model both contain the C3k2 module. To verify the rationality of the improvements made to the C3k2 module, an ablation experiment was conducted, and the comparison results are shown in Table 1.

From Table 1, it can be seen that in Model 2, the Backbone and Neck parts use the FasterBlock module to lightweight the C3k2 module, resulting in the improved module C3k2-Faster. Model 2 has 11.24% fewer parameters and 7.94% fewer GFLOPs compared to Model 1, making Model 2 more lightweight. The introduction of the FasterBlock module reduces the model’s parameter count and computational load, improving the detection speed. However, the precision (P), recall (R), mAP50, and mAP50-95 of Model 2 are reduced by 2.0, 1.2, 0.9, and 2.3 percentage points, respectively, compared to Model 1, indicating a decrease in model detection accuracy. The FPS of Model 2 is 42.9, which is lower than that of Model 1, suggesting that while the lightweight design reduces computation, it also sacrifices some inference speed.

To compensate for the decline in detection accuracy caused by the lightweight design of the C3k2 module, the EMA attention mechanism was introduced on top of C3k2-Faster, creating the improved module C3k2-Faster-EMA, which enhances the model’s ability to focus on small targets and improves detection accuracy. In Model 5, the Backbone and Neck parts both use C3k2-Faster-EMA to replace the C3k2 module. Although Model 5 shows a 0.4 and 0.3 percentage point increase in recall (R) and mAP50 compared to Model 1, it also shows a 0.8 and 0.3 percentage point decrease in recall (R) and mAP50-95, respectively. Additionally, Model 5 has more parameters than Model 1, and its FPS drops to 23.5, making it the slowest in terms of inference speed. Therefore, replacing the C3k2 module in both the Backbone and Neck parts with the C3k2-Faster-EMA module does not yield the best results.

In Model 4, the Backbone part uses C3k2-Faster to replace the C3k2 module, and the Neck part uses C3k2-Faster-EMA to replace the C3k2 module. This model’s precision (P), recall (R), mAP50, and mAP50-95 are 0.3, 2.0, 2.0, and 2.5 percentage points higher than those of Model 5. In Model 3, the Backbone part uses C3k2-Faster-EMA to replace the C3k2 module, and the Neck part uses C3k2-Faster to replace the C3k2 module. This model achieves 75.2%, 72.2%, 77.5%, and 43.2% for precision (P), recall (R), mAP50, and mAP50-95, respectively. The model has fewer parameters and GFLOPs. Although the FPS of Model 3 drops to 30.6, it achieves the highest detection accuracy, providing a good balance between detection accuracy and speed, maintaining a reasonable inference speed. Therefore, this study chooses to improve the YOLOv11 model by using C3k2-Faster-EMA in the Backbone part and C3k2-Faster in the Neck part.

In this study, the lightweight dynamic upsampling operator DySample replaced the original upsampling module UnSample in the Neck, and the improved detection head SEAMHead replaced the original detection head Detect. To validate the effectiveness of the DySample upsampling operator and SEAMHead in improving the YOLOv11n model based on Model 3, ablation experiments were conducted, and the comparison results are shown in Table 2.

From Table 2, it can be seen that incorporating the DySample module into the baseline YOLOv11n model leads to performance improvements across all key metrics. Specifically, precision, recall, mAP@50, and mAP@50:95 increased by 0.3, 0.9, 0.9, and 1.1 percentage points, respectively. Although the number of parameters slightly increased by 0.1 × 10⁵, the GFLOPs remained unchanged, while the FPS significantly increased to 78.4. These results indicate that DySample enhances the quality of feature upsampling and improves inference efficiency without increasing computational complexity. When the SEAMHead is integrated into YOLOv11n, all evaluation metrics—precision, recall, mAP@50, and mAP@50:95—exhibit slight decreases. Nevertheless, both the parameter count and GFLOPs are reduced, and the FPS rises from 48.2 to 70.6. This suggests that SEAMHead optimizes the detection head structure, reduces model complexity, and enhances inference speed, despite a marginal drop in detection performance. Moreover, when both DySample and SEAMHead are jointly integrated into the YOLOv11n framework, precision, recall, mAP@50, and mAP@50:95 improve by 0.5, 1.4, 1.9, and 1.9 percentage points, respectively. Meanwhile, the parameter count increases by only 0.2 × 10⁵, GFLOPs are reduced by 0.5, and FPS increases to 68.9. Compared with incorporating either DySample or SEAMHead individually, the combined approach achieves a more favorable balance between detection accuracy and computational efficiency. These results demonstrate the complementary effects of DySample and SEAMHead, which, together, significantly enhance both the accuracy and real-time performance of the YOLOv11n model.

Based on Model 3, the lightweight dynamic upsampling operator DySample replaced the original upsampling module UnSample in the Neck, and the improved model saw a 0.5, 0.8, 0.6, and 1.4 percentage point increase in precision (P), recall (R), mAP50, and mAP50-95, respectively. Additionally, although the introduction of DySample slightly increased the parameter count, the GFLOPs remained unchanged. However, this improvement led to an increase in FPS from 30.6 to 87.2, indicating that DySample made the model more efficient, contributing to real-time detection tasks. This validates the effectiveness of DySample in the model, as it further optimized the sample point sampling strategy, improving the model’s detection accuracy and speed.

On the basis of Model 3, the SEAM attention mechanism was introduced to improve the detection head, and the improved detection head, SEAMHead, replaced the original detection head, Detect. After the improvement, the model’s precision (P) increased by 0.2 percentage points, but recall (R), mAP50, and mAP50-95 decreased. Compared to DySample, SEAMHead slightly reduced the parameter count, with GFLOPs decreasing to 5.3, and FPS increasing to 81.3. SEAMHead mainly optimized the detection head structure, enhancing the model’s ability to represent features of objects at different scales, while reducing redundant computations. However, the introduction of SEAMHead alone led to a decrease in recall (R), which suggests a possible negative impact on small target detection. Despite this, SEAMHead still reduced computational load, increased inference speed, and made the model more lightweight.

When both DySample and SEAMHead were introduced on the basis of Model 3, the resulting model, FEDS-YOLOv11, saw a 1.6, 1.6, 1.7, and 2.7 percentage point increase in precision (P), recall (R), mAP50, and mAP50-95, respectively. The model’s parameter count decreased by 3.49%, GFLOPs dropped to 5.3, and FPS increased from 30.6 to 71.8, a 134.64% improvement over Model 3. The combination of DySample and SEAMHead compensated for the drop in recall (R) seen when SEAMHead was used alone and further optimized the model’s computational load, achieving a better balance between detection accuracy and inference speed. Both DySample and SEAMHead resulted in superior performance in terms of precision (P), recall (R), mAP50, and mAP50-95 compared to using either of them alone, demonstrating that their synergy maximized the performance of YOLOv11n in forest fire detection.

Comparing the improved FEDS-YOLOv11n model with the original YOLOv11n model, FEDS-YOLOv11n showed a 0.9, 1.9, 2.1, and 2.3 percentage point increase in precision (P), recall (R), mAP50, and mAP50-95, respectively. Its parameter count decreased by 21.32%, GFLOPs decreased by 26.98%, and FPS increased from 48.2 to 71.8, making the model more lightweight and faster in inference. DySample optimized the upsampling strategy, improving the quality of feature map reconstruction and enhancing the model’s ability to distinguish object edges, which, in turn, increased recall (R) and mAP. SEAMHead enhanced the detection head structure, improving the model’s ability to recognize objects at multiple scales, resulting in significant improvements in precision (P), recall (R), mAP, and mAP50-95. DySample reduced unnecessary computations through dynamic sampling, making upsampling more efficient, while SEAMHead employed a more lightweight detection head structure, reducing redundant parameters while maintaining detection accuracy.

In summary, the FEDS-YOLOv11n model, by introducing DySample and SEAMHead, achieves a lower computational cost and faster inference speed while maintaining high accuracy, making it suitable for applications like forest fire detection that require both real-time performance and resource efficiency.

#### 4.4.2. Comparison with Common YOLO Models

To validate the effectiveness of the FEDS-YOLOv11n forest fire detection model proposed in this paper, it was compared with commonly used models such as YOLOv5n [26], YOLOv8n, YOLOv10n [27], and YOLOv11n. The comparison results are shown in Table 3.

From Table 3, it can be seen that the FEDS-YOLOv11n model outperforms other models in both precision (P) and recall (R), with P reaching 76.8% and R being 73.8%, significantly higher than the other models. This indicates that FEDS-YOLOv11n can improve the recognition coverage of fire targets while ensuring the accuracy of the detection results. In comparison, YOLOv5n and YOLOv8n have relatively lower recall (R) values, at 68.6% and 69.4%, respectively, while YOLOv10n and YOLOv11n show some improvement, but still do not reach the performance of FEDS-YOLOv11n. The mAP50 and mAP50-95 of the FEDS-YOLOv11n model reached 79.2% and 45.3%, respectively, which are significantly better than those of the other models. In contrast, YOLOv5n has an mAP50 of 73.9% and YOLOv8n of 73.5%, both lower than those of YOLOv10n and YOLOv11n, while YOLOv11n’s mAP50-95 reached 43.0%, still slightly behind that of FEDS-YOLOv11n. This indicates that FEDS-YOLOv11n performs more stably across different IoU thresholds and has stronger fire target detection capabilities.

The number of model parameters and floating point operations (GFLOPs) are important indicators of model complexity and computational resource requirements. FEDS-YOLOv11n has only 22.1 $\times \;10^{5}$ parameters, which is fewer than the parameters of the other models, indicating that its model structure has been optimized and is more lightweight. At the same time, its GFLOPs is 5.3, the lowest among all models, which indicates higher computational efficiency, making it suitable for deployment in resource-constrained environments. YOLOv8n and YOLOv10n have higher GFLOPs (8.1 and 8.2), making them less suitable for real-time use. Although YOLOv11n shows some improvements, its GFLOPs remain higher than those of FEDS-YOLOv11n. FEDS-YOLOv11n’s FPS is 71.8, the highest among all the compared models. This means that FEDS-YOLOv11n can maintain a high detection speed with relatively low computational costs, making it suitable for scenarios that require real-time performance, such as forest fire detection.

#### 4.4.3. Comparison with Other Mainstream Object Detection Models

To further validate the superiority of the proposed FEDS-YOLOv11n model, a series of experiments were conducted under the same testing environment using several mainstream object detection models. The performance comparison results are presented in Table 4.

From Table 4, the FEDS-YOLOv11n model achieves a precision of 76.8% and a mAP50 of 79.2%, which are significantly higher than those of the other models, demonstrating its superior accuracy in object recognition. Compared with traditional two-stage detectors such as Faster R-CNN and RetinaNet, FEDS-YOLOv11n not only improves detection precision but also maintains lower computational complexity. In terms of model size, FEDS-YOLOv11n contains approximately 2.21 million parameters, making it one of the most lightweight models among the comparisons, surpassed only by EdgeFireSmoke++ and EfficientDet. The substantially smaller parameter count indicates that FEDS-YOLOv11n exhibits stronger lightweight characteristics, making it more suitable for deployment on resource-constrained edge computing platforms or embedded systems. Moreover, the FEDS-YOLOv11n model achieves an inference speed of 71.8 FPS, significantly outperforming other models. In contrast, models like RetinaNet and Faster R-CNN suffer from slower inference speeds due to their complex architectures. FEDS-YOLOv11n effectively balances detection accuracy and real-time performance, making it particularly well-suited for time-sensitive applications such as forest fire inspection tasks.

The results indicate that FEDS-YOLOv11n performs excellently across all performance metrics, especially in recall, mean average precision, model complexity, and inference speed, showing significant advantages and making it more aligned with the actual needs of forest fire detection. Compared to traditional YOLO models, the proposed FEDS-YOLOv11n model has significant advantages in forest fire detection, being a lightweight and high-performance fire detection model that significantly improves detection accuracy and stability.

**Figure 8:**
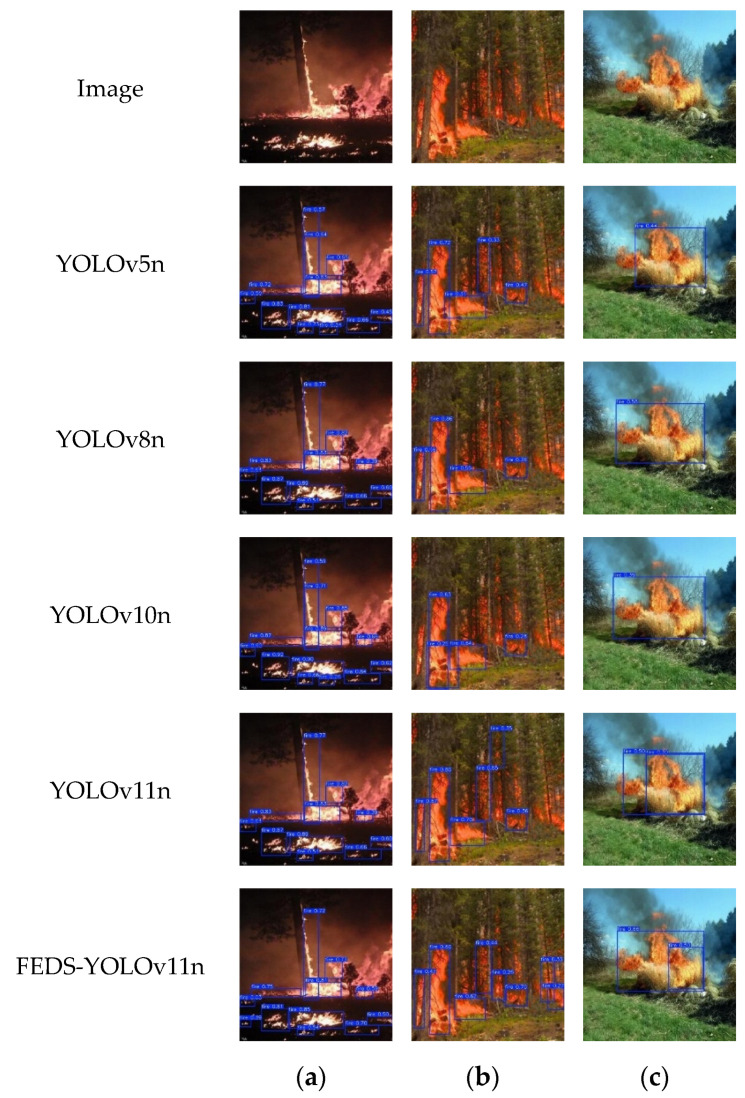
Comparison of forest fire detection results of different YOLO models. (**a**) Detection results for Image (**a**) using YOLOv5n, YOLOv8n, YOLOv10n, YOLOv11n, and FEDS-YOLOv11n; (**b**) Detection results for Image (**b**) using YOLOv5n, YOLOv8n, YOLOv10n, YOLOv11n, and FEDS-YOLOv11n; (**c**) Detection results for Image (**c**) using YOLOv5n, YOLOv8n, YOLOv10n, YOLOv11n, and FEDS-YOLOv11n.

The comparison of forest fire detection results from different YOLO models is shown in Figure 8.

From Figure 8, it can be observed that the YOLOv5n, YOLOv8n, YOLOv10n, and YOLOv11n models all demonstrate strong detection performance in forest fire detection tasks, but they have certain limitations when dealing with small target flames and complex backgrounds. As shown in the detection results of each model in Figure 8a,b, the YOLOv5n, YOLOv8n, YOLOv10n, and YOLOv11n models all exhibit missed detection for small target fires, and their detection performance on small targets is not ideal. In contrast, the improved FEDS-YOLOv11n model can still effectively detect small targets, even in cases where the flame area is small or the flame color is similar to the background. As seen in the detection results in Figure 8c, for the same flame, the detection probability distribution of the YOLOv5n, YOLOv8n, YOLOv10n, and YOLOv11n models is relatively low, while the detection probability of flames in the FEDS-YOLOv11n model is significantly higher than that of the other models, demonstrating stronger fire target recognition ability. In comparison, the improved FEDS-YOLOv11n model, by incorporating feature enhancement modules and attention mechanisms, focuses more on the key features of the flame, effectively improving detection accuracy in small target and complex scenarios. Additionally, the model performs better in detecting fine details at the edges, reducing the blurriness of the flame-background boundary and effectively lowering the rates of missed and false detections.

Overall, the FEDS-YOLOv11n model demonstrates the best performance in forest fire detection tasks, particularly for small target flame detection and complex fire environments. Its detection accuracy and real-time performance are significantly better than those of YOLOv5n, YOLOv8n, YOLOv10n, and the original YOLOv11n model.

## 5. Conclusions

Existing forest fire detection models still have certain shortcomings in detection speed, accuracy, and resource utilization, especially in application scenarios with small target detection and high real-time requirements. To address this, this paper proposes a lightweight, high-performance fire detection model, FEDS-YOLOv11n, based on the YOLOv11n model. The C3k2-Faster module replaces C3k2 in the Backbone and Neck sections to achieve a lightweight design. C3k2-Faster-EMA replaces C3k2-Faster in the Backbone, compensating for the reduced small target detection accuracy caused by lightweight design. A lightweight dynamic upsampling operator, DySample, replaces the original upsampling module in the Neck. The improved detection head SEAMHead replaces the original Detect head, enhancing the model’s ability to handle occluded targets. The experimental results show that, compared to the original YOLOv11n and other commonly used YOLO models, the FEDS-YOLOv11n model demonstrates significant advantages in detection accuracy, recall rate, model complexity, and computational efficiency, offering lower computational complexity and higher inference speed while maintaining high precision, making it more suitable for fire detection in complex forest environments.

Although FEDS-YOLOv11n performs excellently in forest fire detection, there is still room for further improvement in future research. First, to address the dynamic changes in fire environments, the combination of temporal information can be explored to improve adaptability to dynamic fire scenarios. Second, although FEDS-YOLOv11n has achieved good results in small target detection, it still faces challenges from diverse targets and complex backgrounds that may occur in different fire scenarios. Future work may consider more data augmentation techniques and model adaptation optimization methods. Finally, although the model has not yet been deployed on an actual embedded system, its lightweight architecture and fast inference performance provide a solid foundation for integration into the NVIDIA Jetson Nano platform. Future research will focus on accelerating inference on low-power devices and conducting field tests in real forest environments.

## Figures and Tables

**Table 1:** Results of different C3k2 improvement methods.

Model	BackBone	Neck	P/%	R/%	mAP50 /%	mAP50-95 /%	Params $\times 10^{5}$	GFLOPs	FPS
1	C3k2	C3k2	75.9	71.9	77.1	43.0	25.8	6.3	48.2
2	C3k2-Faster	C3k2-Faster	73.9	70.7	76.2	40.7	22.9	5.8	42.9
3	C3k2-Faster-EMA	C3k2-Faster	75.2	72.2	77.5	43.2	22.9	5.8	30.6
4	C3k2-Faster	C3k2-Faster-EMA	74.6	69.1	75.4	40.2	22.9	5.8	31.6
5	C3k2-Faster-EMA	C3k2-Faster-EMA	76.3	71.1	77.4	42.7	29.3	5.9	23.5

**Table 2:** Comparison of results of improved ablation experiments with YOLOv11n model.

Model	P/%	R/%	mAP50/%	mAP50-95/%	$\mathrm{Params}\times 10^{5}$	GFLOPs	FPS
YOLOv11n	75.9	71.9	77.1	43.0	25.8	6.3	48.2
YOLOv11n + DySample	76.2	72.8	78.0	44.1	25.9	6.3	78.4
YOLOv11n + SEAMHead	76.0	71.2	76.8	42.3	24.9	5.8	70.6
YOLOv11n + DySample + SEAMHead	76.4	73.3	79.0	44.9	25.0	5.8	68.9
Model 3	75.2	72.2	77.5	43.2	22.9	5.8	30.6
Model 3 + DySample	75.7	73.0	78.1	44.6	23.0	5.8	87.2
Model 3 + SEAMHead	75.4	70.8	76.7	41.8	22.0	5.3	81.3
Model 3 + DySample + SEAMHead	76.8	73.8	79.2	45.3	22.1	5.3	71.8

**Table 3:** Comparison of results of different YOLO models.

Model	P/%	R/%	mAP50/%	mAP50-95/%	$\mathrm{Params}\times 10^{5}$	GFLOPs	FPS
YOLOv5n	73.3	68.6	73.9	38.5	25.0	7.1	54.4
YOLOv8n	71.9	69.4	73.5	39.2	30.1	8.1	64.8
YOLOv10n	72.7	71.0	74.2	40.6	26.9	8.2	41.9
YOLOv11n	75.9	71.9	77.1	43.0	25.8	6.3	48.2
FEDS-YOLOv11n	76.8	73.8	79.2	45.3	22.1	5.3	71.8

**Table 4:** Comparison with other mainstream object detection models.

Model	P/%	mAP50/%	$\mathrm{Params}\times 10^{5}$	FPS
SSD	68.2	72.1	241.2	47.2
Faster R-CNN	72.3	75.8	413.8	12.5
Fire-Net	71.5	74.0	85.2	40.1
RetinaNet [28]	73.0	76.3	339.6	22.4
EfficientDet [29]	70.5	75.2	39.3	34.6
EdgeFireSmoke++	74.4	76.0	33.5	52.3
FEDS-YOLOv11n	76.8	79.2	22.1	71.8

## Data Availability

The data presented in this study are available upon request from the corresponding author. The dataset and code cannot be shared due to specific reasons.
